# Lifestyle behaviour change following breast cancer: A qualitative exploration of experiences and unmet support and information needs

**DOI:** 10.1177/13591053251336843

**Published:** 2025-06-11

**Authors:** Lauren Matheson, Lucy McGeagh, Julie Bennett, Emma L Davies, Jo Brett, Sara Matthews, Eila Watson

**Affiliations:** Oxford Brookes University, UK

**Keywords:** alcohol, breast cancer, diet, exercise behaviour, lifestyle, qualitative methods

## Abstract

Lifestyle modification can reduce breast cancer recurrence risk and improve quality of life. However, few studies have explored patients’ experiences of lifestyle behaviour changes following breast cancer, specifically the information and support received regarding lifestyle changes. To explore this topic, interviews were conducted (*n* = 21 women) and analysed thematically based on the Framework Approach. Themes included: *Impact of cancer and behaviour change complexities; Impact of lifestyle messaging from healthcare professionals; Desire for empowering lifestyle advice* and *Shaping future lifestyle interventions*. Lifestyle advice was either not provided, or perceived as unhelpful, with some being discouraged from making changes during treatment. If advice was received, emphasis tended to be in relation to physical activity and diet, with little mention of alcohol consumption. Personalised and empowering interventions are needed for patients and healthcare professionals to increase awareness regarding benefits of lifestyle changes after breast cancer, particularly regarding alcohol reduction, and to promote behaviour change.

## Introduction

Breast cancer is the most common cancer in the UK (Cancer Research UK, 2024) with incidence continuing to rise. Despite improved rates of 10-year survival at around 75% (Cancer Research UK, 2024), recurrence remains a risk, occurring in approximately 20% of survivors (Early Breast Cancer Trialists’ Collaborative Group (EBCTCG), 2018). Breast cancer survivors may experience a range of treatment-related side effects which can impact their quality of life (QoL), including pain, decreased strength, lymphoedema, body image disturbance, weight gain, anxiety, depression and fatigue (Lovelace et al., 2019; Matias et al., 2019; King et al., 2024; Vance et al., 2011). Lifestyle modifications have a range of positive impacts on breast cancer outcomes. These include reducing recurrence and mortality, improving QoL, and managing the consequences of treatment (Kennedy et al., 2023; Montagnese et al., 2021; Tsilidis et al., 2023; Yeganeh et al., 2024).

### Physical activity

The World Cancer Research Fund (WCRF, 2018; WCRF CUP Global, 2024), recommends that breast cancer survivors should aim to be physically active and avoid obesity. Evidence illustrates that physical activity can reduce breast cancer mortality by 34% and recurrence by 24% (Cariolou et al., 2023; Lahart et al., 2015; Tsilidis et al., 2023). In addition, physical activity can help manage the consequences of cancer and treatment, and lead to improved QoL (Kennedy et al., 2023). The WCRF advises breast cancer survivors to follow the same guidance as the general population (i.e. at least 150 minutes of moderate exercise and two sessions of resistance exercise per week; WCRF, 2018; World Health Organisation, 2024). Psychosocial benefits have been found in women following breast cancer treatment who exercised at these levels for 6 months (Pinto et al., 2013). Further to this, there is strong evidence for the association of higher Body Mass Index (BMI) with all-cause and breast cancer mortality and growing evidence for association with recurrence (Chan et al., 2023).

### Alcohol

Evidence regarding the impact of alcohol consumption on breast cancer survivors is less established, in comparison to other lifestyle factors (Kwan et al., 2023; Terry et al., 2021). However, there is a known link between alcohol intake and the development of breast cancer, with 98,300 new breast cancer cases globally being attributed to alcohol consumption in 2020 (Rumgay et al., 2021). Daily alcohol consumption has been associated with increased risk of late recurrence (Nechuta et al., 2016; Terry et al., 2021). WCRF (2018) report that drinking even one alcoholic drink a day can increase risk of breast cancer. It is therefore recommended that breast cancer survivors limit alcohol intake to fewer than 5 units of alcohol per week or avoid alcohol altogether (NICE, 2024; WCRF, 2018). In the UK, 15% of women in the general population are drinking at high risk levels (over 14 units), with 30% in mid-life drinking at the highest levels, compared to any other age group (NHS England, 2024). There is also a lack of awareness regarding the association between alcohol and breast cancer in the general UK population (Davies et al., 2024a). While international data on breast cancer survivors suggests that almost a third consume alcohol at risky levels (DeNysschen et al., 2015), there is a lack of any research to date focused on alcohol-related behaviours amongst UK survivors.

### Diet

The WCRF also suggests dietary recommendations for breast cancer survivors (WCRF, 2018). This includes eating wholegrains, vegetables, fruit, avoiding sugary drinks and processed meats or food high in fats, starches or sugars, and limiting red meat consumption. However, existing research for dietary guidance following breast cancer is limited (WCRF, 2018). Evidence so far suggests that increased fibre and isoflavone (found in soy) could reduce breast cancer mortality (Becerra-Tomás et al., 2023). There has been some exploration of dietary behaviours in breast cancer survivors highlighting barriers to making healthy food choices (Keaver et al., 2021; Kwok et al., 2015). However, few studies to date have explored this in the UK.

Adherence to the WCRF guidance, and additionally, avoiding smoking, has been found to improve QoL and reduce fatigue in mixed groups of cancer survivors (Kennedy et al., 2023). Although it is uncertain how widely guidelines are known or adhered to in the UK breast cancer population, some targeted interventions to support such individuals to adopt healthier lifestyles have been developed. Theory-based interventions for behavioural change are effective at increasing physical activity (Bluethmann et al., 2017; Hailey et al., 2022; Liu et al., 2022). Understanding behaviour change through frameworks such as the Capability, Opportunity, Motivation and Behaviour (COM-B) model could aid the development of future interventions (Ee et al., 2022). However, further work to inform intervention development is required, in order to better understand lifestyle behaviour change in this population.

It is important that breast cancer survivors are supported to have a healthy lifestyle, to improve wellbeing and QoL. However, as outlined above, few studies have explored the experiences of breast cancer survivors in relation to making changes to alcohol consumption, diet or physical activity or their support and information needs regarding lifestyle (Hardcastle et al., 2019; Kim et al., 2020; Ko et al., 2023). This study therefore aimed to explore experiences of making lifestyle modifications and views towards the lifestyle behaviour support and information received since their breast cancer diagnosis. We have focused particularly on alcohol consumption, due to the dearth of research on this topic. To inform intervention development, we also aimed to explore views regarding future interventions to promote healthy lifestyle behaviour change.

## Methods

### Design

Qualitative interviews were conducted as part of a larger mixed-methods study that included an online survey. Feedback from three Patient and Public Involvement (PPI) members (breast cancer patients) were incorporated into the overall study design. They suggested additional recruitment pathways via online forums, amended the interview topic guides from primarily focussing on alcohol to include all lifestyle behaviours, and revised patient facing documentation.

The full study protocol was registered on the Open Science Framework.^1^ Ethical approval was granted by Oxford Brookes University (ref 211553). All participants provided written informed consent to participate. Consolidated criteria for reporting qualitative research (COREQ) guidelines have been used (see Supplemental File 1; Tong et al., 2007).

Mixed-methods findings focussing solely on alcohol behaviour changes have previously been reported (Davies et al., 2024b) and general findings from our online survey will be reported (Brett et al., 2023).

### Recruitment

Interview participants were recruited through the online survey. For the survey, reported elsewhere (Davies et al., 2024b), participants recruited were 18 years and over, UK residents, of any gender, and within 10 years of completing primary breast cancer treatment. Survey participants were recruited via social media (X, Facebook groups), through existing patient contacts, cancer support groups, alcohol support organisations or breast cancer charities.

At the end of the survey, participants were asked if they would be interested in taking part in an interview. Of the 140 survey participants, 79 agreed to be contacted about an interview. An email invitation was sent to 25 of these participants; purposively selecting people who indicated that they had changed their drinking (either increased or reduced). This was to understand more about alcohol consumption in those who were/had been drinkers, and to address the gap in the literature on this behaviour.

### Interviews

The interviews followed a semi-structured topic guide (see Supplemental File 2), developed using data from the survey, PPI input, literature and the research questions. Topics focused on experiences of lifestyle behaviour changes since diagnosis, particularly experiences of receiving lifestyle information and support, and views towards potential lifestyle interventions. Interviews were conducted by four experienced qualitative researchers, LMcG, JB, LM and JBr (all female, with psychology and/or applied health, not clinical, research backgrounds) and were audio recorded. Reflective notes were kept, and a summary written following each interview, which was shared with the study team.

### Analysis

Thematic analysis based upon the Framework Approach (Gale et al., 2013) was employed, and NVivo software assisted with coding and managing the data. Analysis involved reading through the transcripts to increase data familiarity. Three transcripts were then coded by two researchers (LMcG and LM) for items of data relating to the research question, and initial codes generated. These were discussed with the full team. An initial coding framework (Supplemental File 3) was then developed by one researcher (LMcG). This was discussed and revised with a second researcher (LM), and then reviewed by the study team, which was an iterative process. Transcripts were then coded independently by three researchers (LMcG, LM and SM) using the developed framework. Preliminary themes were generated, which were reviewed and refined until a coherent narrative of the participants’ experiences was produced through four core themes.

## Results

### Participants

Of the 25 invited to take part in an interview, 21 agreed to participate, and the remaining four did not respond. Data saturation (Guest et al., 2020; Hennink and Kaiser, 2022) was reached after the 21 interviews were completed. Interviews, conducted between October 2022 and April 2023, lasted for an average of 63 minutes (range 28–100 minutes), and were conducted either via Zoom video call (*n* = 12) or telephone (*n* = 9). Participant characteristics are presented in Table 1. All interview participants self-identified as being female. Women interviewed had received a breast cancer diagnosis between 2013 and 2021, all were White, and over half were aged 50–59 years (*n* = 11, 52%). There was a fair geographical UK spread.

**Table 1.** Characteristics of interview participants.

ID	Year of diagnosis	Age	Education	Locality	Self-reported health^a^
1	2021	60–69	A levels	South Central England	Fair
2	2021	50–59	Postgraduate	South East England	Very good
3	2020	50–59	other	South West England	Excellent
4	2014	40–49	Undergraduate	Wales	Very good
5	2021	50–59	GCSE/O levels	South Central England	Good
6	2020	50–59	Postgraduate	North West England	Good
7	2018	50–59	Undergraduate	Scotland	Very good
8	2016	50–59	Undergraduate	Wales	Very good
9	2020	50–59	A levels	South East England	Very good
10	2020	50–59	Postgraduate	North East England	Very good
11	2018	60–69	Postgraduate	West Midlands	Fair
12	2016	60–69	other	Yorkshire and Humber	Very good
13	2018	50–59	Postgraduate	Wales	Very good
14	2015	40–49	Postgraduate	South East England	Good
15	2021	50–59	A levels	Scotland	Very good
16	2013	40–49	Undergraduate	West Midlands	Excellent
17	2014	60–69	GCSE/O levels	South East England	Very good
18	2021	60–69	Postgraduate	North West England	Very good
19	2019	50–59	Postgraduate	East Midlands	Very good
20	2018	60–69	Undergraduate	North East England	Very good
21	2015	60–69	A levels	East Midlands	Very good

a Data collected from our survey (Davies et al., 2024b).

Below we present the four core themes. Table 2 (Supplemental File 4) summarises the themes; additional exemplar quotes are provided in Tables 3–6 (Supplemental Files 5–8).

### Theme 1: Impact of cancer and behaviour change complexities

This theme relates to the lifestyle changes and new habits that participants described, including the reasons why they made changes, and other contextual reasons that contributed to this.

(a) Formation of new healthier habits

Most women reported lifestyle changes they had made following a diagnosis of breast cancer, which often appeared to reflect motivation for a reprioritisation of their health. Changes described included adopting new exercise regimes and habits, such as starting to run, swim or cycle, participating in group exercise classes and generally being more physically active, with women perceiving many benefits to these new behaviours. Some also reported adopting a healthier, more balanced diet, as some had used new recipe books, or adopted better habits such as cooking from scratch. Some also discussed reducing alcohol intake.

I changed my hobbies, my exercise regime, the way I ate, everything… I eat way more healthy… it was only after my diagnosis that I started to really look into a balanced diet, and a diet that wasn’t based on convenience food… (P4)

Some participants attributed behaviour changes to circumstances beyond their cancer (or indirectly affected by it). These included changes in work patterns or careers, such as retirement, which were facilitators to having more time to do physical activity or cook healthy meals. Changes to alcohol consumption were sometimes attributed to other events, such as the menopause or ageing.

(b) Reasons for lack of behavioural changes

Some women did not report any lifestyle behavioural change – often this was because they perceived themselves to have been healthy before their diagnosis. In relation to alcohol, some reported drinking less, particularly during active treatment, but also as an ongoing lifestyle change. However, this was not the case for all women, with some resuming their normal levels of drinking after treatment, not wishing to permanently reduce consumption. Whilst many complex barriers to alcohol reduction were indicated (described in detail elsewhere (Davies et al., 2024b), this was often due to women not associating alcohol with their breast cancer risk, and/or their risk of recurrence. This lack of awareness was particularly evident regarding alcohol amongst other lifestyle behaviours.

I’m thinking well the odd glass of red wine has not given me breast cancer, and I’m not gonna cut it out of my life, and especially now, life’s too bloody short not to have a drink of a nice red wine… I certainly haven’t changed my drinking habits… (P16)

As discussed further in Theme 2, this lack of awareness was impacted by a general deficit in advice received on alcohol consumption by healthcare professionals (HCPs), so some women did not perceive a need to change this behaviour. Some women talked about contextual reasons for not making health behaviour changes in other areas. This included, having to feed a family of fussy eaters hampering healthy eating for themselves, or having confounding medical conditions for example, pain, or side effects from endocrine therapies, which impacted physical activity levels.

It isn’t as easy or as enjoyable as it used to be. I can’t do the distance anymore, my joint pain really inhibits the distance that I can do and how comfortable it is when I’m actually walking. (P19)

### Theme 2: Impact of lifestyle messaging from healthcare professionals

This theme relates to the impact of lifestyle messaging from HCPs (any advice, information and/or support given) on participants’ beliefs and behaviours regarding their lifestyle.

(a) No lifestyle advice received

Some women talked about not receiving any lifestyle advice or recommendations from HCPs at any point.

They [HCPS] don’t give you any advice. They don’t say anything, or they’ll tell you not to do something, they don’t give you active advice as to what you can do (P16)

This applied to all lifestyle behaviours, including diet and physical activity advice, but the gap in advice seemed particularly evident regarding alcohol reduction. Many were surprised or disappointed, that this support or information was not provided, given their general positive experience of National Health Service (NHS) care. Some women did acknowledge however, that the information may have been given, and they may have forgotten.

I think there’s so many missed opportunities to link in with you. And you may not be receptive to it, and some people may not be interested… but I think having the access to it, giving it to people. Because I had to physically find things… NHS is so busy; they do a brilliant job… and where it falls through the cracks is not intentional. (P10)

Some described seeking out individualised lifestyle advice or information, often from sources outside of the NHS for example, books, celebrity cancer survivors via social media, personal trainers or nutritionists and cancer charities, which were described as sometimes incurring personal costs to fund.

(b) Weak advice

Other women perceived that where advice had been given by HCPs; it had been weak or lacked conviction. At times, it was felt that advice given was too generic, for example *‘Just try to be relatively healthy’* (P9), rather than personalised to them.

It was just very wishy-washy. Yeah, if you fancy a drink you can have one… (P13)

It was suggested by some that HCPs placed much less importance, if any, on imparting lifestyle advice, compared to medical information.

It’s an afterthought, it’s an ad hoc, it’s a bit of a it’ll do you good to have a walk and not drink or blah blah, eat more carrots, whatever. It’s an afterthought, it’s not a priority, it’s not perceived, and so you don’t put it as a priority (P20)

There were some women who felt that HCPs needed to be more direct (see Theme 3), explicitly highlighting that there could be links between lifestyle behaviours, cancer diagnoses and future recurrences. It was suggested by a few that the type of advice given was often reliant on the personal views of HCPs.

(c) Unhelpful self-care advice in lifestyle messaging

There was a sense that some lifestyle messaging had been unhelpful and potentially led women to be less healthy. For example, being advised not to stop smoking or cut down alcohol intake during treatment as this could add additional stress at an already challenging time. It may have been that HCPs were suggesting patients practice self-care, in the form of ‘taking it easy’. However, in a few instances this advice appeared to support sedentary behaviours or potentially reinforced unhealthy lifestyles. Advice from some HCPs prior to or during early treatment included discouragement of particular types of physical activity.

I remember saying to her [chemo nurse] I’m still planning to run, and she’s like well you won’t be able to do that. So there was a lot of people saying that I wouldn’t be able to (P2)

One participant wished she had been encouraged to do more at the start of her treatment, especially because as soon as treatment ended she was then told to increase her physical activity levels. However, by this point her sedentary lifestyle had led to further health implications.

My primary takeaway from that time [treatment] was don’t overdo it, eat what you want, .. somebody said if you smoke don’t give up now, if you drink excessively, don’t give up now…. I get that . . . the purpose of it was don’t put additional stress on yourself. Because you’re going to have a stressful time… I just thought the message was wrong… I wished I’d been told… Been encouraged a bit more… And then as soon as you finish that treatment I remember being told… what you need to do now is do squats while you’re on the telephone… And I put on two and a half stone in 12 months… it could have been avoidable… (P4)

(d) The salience and impact of a passing comment

For alcohol advice in particular, it appears that at times potentially harmful messages were communicated by HCPs, which were not in line with current recommendations or had a strong evidence base.

There was a nurse at the hospital …And she also said take vitamin D, before bed every night… so I’ve taken vitamin D every day for about eight years, my husband as well (P17)

Several examples were given, whereby women described a single comment made by a HCP, (often one made in passing), which although may not have been intended to have a large impact, held significance for the women. Advice from HCPs s could be strongly weighted particularly where a trusting relationship existed.

I checked in with one doctor… and I said .. can I talk to you again about the drinking? And he said to me are you a heavy drinker? And I said [described occasional binge drinking]… and he said well that that’s not heavy drinking, that’s life, and ..he very much played it down… Normalising it (P14)

The following participant who described heavy drinking prior to her cancer diagnosis, reported giving up drinking following diagnosis; however, a passing comment by her consultant ‘gave her permission’ to return to drinking. While it is very unlikely that this was the consultant’s intention, this was how the message was interpreted, resulting in a return to high levels of alcohol consumption.

It was when I went to see the consultant .. he’s telling me the good news, and he said you can go home and have a big bottle of champagne now.. it was like a big tick in a box, I can go drink… (P12)

In others, there was confusion and miscommunication surrounding advice from their HCPs, which could potentially do more harm than good. For example, one participant believed they were advising her to cut down on the amount of wine she drank, not on her alcohol levels generally, therefore, she followed their advice and reported that she drinks ‘*gin now instead of red wine*’ (P17). A few women talked about how they found comments made by their clinician to be upsetting; once again these were most likely passing comments, which had an unintended impact.

My oncologist told me I was a big lady and I needed to watch my weight (P11)

For example, this quote demonstrates how something supposedly said to motivate a woman to lose weight, left her feeling quite negative.

### Theme 3: Desire for empowering lifestyle advice – Valuing ‘a strong call to arms’

This theme focuses on women repeatedly noting that they would value clear lifestyle advice at various times during their cancer trajectory.

(a) The value of clear lifestyle advice

As described above, while women were very positive about their healthcare team generally, many wanted more information, guidance and support about making positive lifestyle changes after breast cancer. Those who had received advice, often via sources outside of the NHS, felt that knowledge of how to reduce their risk of recurrence through lifestyle modifications helped motivate them to change, and feel more in control of their lives generally.

I’m quite obsessed about reducing as many risk factors as I can of it coming back, because I am high risk of it coming back… in terms of doing some control over doing what I can to help it not come back, and obviously feeling healthier (P10)

Women reported welcoming clear messages about the need to make lifestyle changes and found this advice to be empowering. For example, participant 10 appreciated the recommendation, via information by a well-known breast cancer survivor, that it would be beneficial if she pushed herself physically to do exercise, even during cancer treatment.

During treatment I did quite a lot of research, and my “Bible” is the [well known healthcare professional/cancer survivor’s] book… .. which basically said exercise every day for a minimum of half an hour during treatment: even if you’re physically sick, do it. It was quite a strong call to arms for me, something I could focus on, had control over (P10)

Further to this, another participant suggested strong advice that was backed up by evidence would be useful ‘show [patients] the outcomes of studies’ (P17).

Exercise, the healthy eating and lifestyle and stress management, I’m absolutely acutely aware of everything I need to be doing, that’s through my own research… So I probably know it more than a lot of people, I still can’t do it, and I don’t know what it is, .. the time factor or… But .. I’m not putting into place all of those factors that I know would help prevent that (P19)

However, as the previous quote suggests, this knowledge alone was not always sufficient to prompt changes, with many other potential barriers, including a lack of time for instance.

(b) The timing and delivery of desired advice

Some women felt regretful they had not received advice on the benefits of lifestyle changes earlier on in their treatment trajectory. These women were aware that physical activity could have helped them to avoid treatment-related weight gain, reduce fatigue and avoid loss of fitness. While some would have wanted this advice soon after or at the time of diagnosis, others suggested this information would be preferred after active treatment. However, it was also discussed that there may not be an ideal time, and that information given at different time points would be welcomed.

Right at the start, because they’re even talking now about the benefits of getting you fit before you’ve even started any treatment, to get you in a better place to withstand the chemotherapy, to be able to recover from your surgery. … I think the exercise message needs to be in there right at the beginning (P16)

Some women felt that a greater emphasis should be placed on these ‘lifestyle treatments’ (P20) and that this message should be ‘completely without judgement’ (P7). Women also wanted information presented to them directly, in a considered and sensitive manner with support offered.

I like people to be honest, and all through my treatment I would rather the doctors be honest. But I’m that kind of person… I’d rather know… and then it’s my choice whether I take that info or not really. I wouldn’t be offended (P8)

Where lifestyle advice was received, some felt that information on alcohol, and the links with cancer (or cancer recurrence), was often missing. Women stated that they would have been receptive to information about current recommendations enabling them to make an informed choice about whether, or not, to reduce consumption.

### Theme 4: Shaping future lifestyle interventions

This theme relates to how women thought unmet needs regarding lifestyle-related support and information could be addressed by future lifestyle interventions.

(a) Multiple modes of delivery for a ‘one stop shop’ intervention

Women were generally very positive towards the need for future lifestyle interventions, both for themselves and others with breast cancer. While digital interventions were preferred by some, having different modes of intervention delivery (face-to-face, digital, written etc) was deemed important. Women described how changes to diet and physical activity often went hand-in-hand, with one behaviour change often prompting another.

It was just when you start to do a little bit more exercise you then want to eat clean, and then once you eat clean… you feel more healthy (P4)

Therefore, it was suggested that having a ‘one stop shop’ (P16) resource for all lifestyle behaviours together would be helpful; they were generally not keen on having a standalone alcohol reduction intervention. Participants also thought that this could be provided alongside mental health support or signposting to further resources.

(b) Personalised support from a trustworthy source

Participants discussed the need for reliable and trustworthy sources of lifestyle information such as scientific research, which for some women, would include statistics highlighting the current evidence base. This could include evidence of the benefits, and the links between lifestyle and cancer. Women felt strongly that advice was not generic but personalised to them. Some also mentioned the benefit of knowing personal risk of recurrence information.

I mean maybe include some statistics… because I know that people think oh god, statistics, but … the most powerful meeting I had was with my oncologist, when we went through all the different things about how likely it was my cancer would come back and all that, and I found that really powerful (P1)

In terms of tailoring, it was also deemed important that interventions consider the barriers specific to breast cancer survivors, such as restricted physical ability or strength. Other barriers described included a general lack of confidence following diagnosis, and a sense of health fatalism towards whether making future changes would make any difference. Therefore, it was emphasised that interventions need to be individually tailored, taking these into consideration. Goal setting regarding lifestyle changes was also thought to be a key component of such an intervention, as was having individualised feedback and monitoring to provide ongoing support with behaviour changes.

It’s that reassurance that if you input something…going for a half hour walk every day and coming back feeling hot and sweaty is… is what I’m doing amazing, or is what I’m doing actually that’s really only just enough. And that’s in relation to all three elements: diet, exercise and alcohol. It’s somebody saying yes, you’re doing more than enough, or actually no you’re not (P13)

Women thought that there should be involvement or input from HCPs as a trustworthy source, as part of the intervention. They were however unsure of the type of professional who would have capacity and/or expertise in both lifestyle behaviours and cancer. This input was suggested to either be through face-to-face, or remote delivery for example, through online videos of HCPs.

I think what might have helped across all those three areas would be to be able to have a chat with someone, who had good knowledge but also was kind of understanding about how advice might make you feel. .. I mean I think with alcohol… if it would really really make a massive difference to my likelihood of recurrence, somebody tells me that straight out that that is gonna make me really have to think very carefully about what I’m doing (P2)

Some suggested nurses, radiographers, rehabilitation physiotherapists or nutritionists who might deliver this advice or information which could be ‘almost like a post-cancer MOT’ (P19).

(c) Social support and emphasis on the benefits of lifestyle changes

Women found physical activity to have many benefits, not only with regards to relaxation, stress management and mental health, but also, with group exercise, as providing social support. Therefore, many discussed the importance of including elements of social support in a future intervention. Peer support from others with cancer was deemed crucial as a way of providing mutual encouragement. Some wanted this in the form of a group, while others thought one-to-one buddying would be preferable, or links to existing local support groups.

Encouragement, group support …. And help on getting the right advice at the right time I think (P11)

It was also perceived as important that the benefits of healthy lifestyles are highlighted by a future intervention, as a way of encouraging others.

## Discussion

Our qualitative study shows that people affected by breast cancer would value receiving personalised and empowering lifestyle-related support and information, with a clear message, delivered in a timely way across the diagnosis, treatment and follow-up trajectory. Some women reported receiving no lifestyle advice from HCPs, or those that did, perceived that the advice was weak, ineffectual and sometimes unhelpful, and not in line with their expectations. This finding is important as women appeared to attach significance to passing remarks regarding lifestyle changes made by HCPs, suggesting that they have an influencing role in patients’ behaviours and awareness regarding the importance of lifestyle. This echoes findings from a previous study with cancer survivors (Hoedjes et al., 2022).

There is clearly a need for future interventions targeted at people with breast cancer, and future interventions or training targeted towards HCPs. Participants had differing preferences as to the timing of this information and support, highlighting that advice may need to be provided at several key points, and individually tailored (Public Health England, NHS England and Health Education England, 2016). While some participants expressed a need for a strong message, HCPs need to be aware of different individual coping styles and responses to advice. Following a cancer diagnosis there may be many opportunities to engage in a dialogue, even if brief, about lifestyle behaviour change – indeed this time is recognised as a potential ‘teachable moment’ for behaviour change (Di Meglio et al., 2021). Little, or no, emphasis was placed on alcohol reduction by HCPs, with only some women making changes in this area. Our wider mixed-methods study (Davies et al., 2024b) has described in depth the key barriers to alcohol reduction, which included drinking as a habitual behaviour and perceptions of the benefits of alcohol, including stress reduction, enjoyment, increased confidence and drinking as a core element of socialising in the UK. This was also echoed in our study of mid-life women in the general population, who were not often aware of the links between cancer and alcohol (Davies et al., 2024a).

Participants in our study highly valued peer support, emphasising the importance of including group and/or one-to-one peer support in a future lifestyle intervention. Peer matching or buddying with similar others was suggested as motivating to them, particularly in regard to physical activity. Existing interventions that include peer support have shown promising findings (Murray et al., 2023), with social support being a key determinant of positive lifestyle change generally (Hoedjes et al., 2022; Park et al., 2008). Our study also supports the need for multi-component approaches to lifestyle, as these behaviours are often clustered together (Fernandes et al., 2023; Noble et al., 2015). For instance, unhealthy lifestyle behaviours (physical inactivity, unhealthy diet, excessive alcohol and smoking) often co-occur together, similarly to healthy lifestyle behaviours (Noble et al., 2015). Therefore, interventions targeted at multiple behaviours are potentially more impactful (Prochaska and Prochaska, 2011).

### Recommendations for practice and future research

Our quantitative data (Davies et al., 2024b) show that few patients are aware of the current WCRF recommendations of no more than 5 units of alcohol per week for cancer survivors (WCRF, 2018). This is unsurprising considering our qualitative findings highlighting the lack of advice on alcohol, as well as the potentially unhelpful advice or comments received from HCPs. However, there are currently no guidelines for HCPs on what breast cancer patients should be told, either during treatment or afterwards. Considering the salience of remarks by HCPs to patients, and the importance of their advice, as demonstrated previously (Hoedjes et al., 2022), it is critical that information patients receive is accurate (and helps them avoid misinformation received from online sources). The wider literature suggests that HCPs may avoid discussing sensitive lifestyle topics if they are concerned it may damage trust and rapport (Koutoukidis et al., 2018). Guidelines and training for HCPs are needed to increase skills, knowledge and confidence to discuss alcohol reduction in a supportive, sensitive and empowering way. Further research needs to consider how best to educate HCPs on the evidence base surrounding a range of lifestyle behaviours.

In addition, our findings highlight that a multi-component intervention that encompasses all key lifestyle behaviours (a ‘*one stop shop’*), rather than on alcohol reduction alone would be preferred. There may also be difficulties with the stigma associated with alcohol reduction interventions (Keyes et al., 2010). However, interventions so far have largely focused on physical activity (with or without diet), without inclusion of alcohol reduction (Carmack et al., 2021; Gnagnarella et al., 2023; Leske et al., 2024). Future interventions developed for a UK setting, need to be cognisant of current challenges faced by the UK NHS oncology workforce, including insufficient clinical nurse specialists in key roles, and time burdens on staff. While supporting patients with lifestyle changes is considered part of the role of the nurse in particular (Thompson, 2019), a survey of 78 multi-disciplinary UK HCPs showed that while almost all agreed lifestyle advice was important, a lack of time, training and detailed recommendations were perceived to be key barriers to implementing this (Whittaker et al., 2024). Innovative ways of supporting lifestyle modifications are needed that do not add additional burden to NHS staff, such as through digital interventions which HCPs can signpost to. Some digital interventions including apps or text messaging interventions, alongside support, have been developed to date, and appear to be largely acceptable to patients (Singleton et al., 2022; Smith et al., 2024). Theoretically informed interventions, that incorporate behaviour change techniques and employ wearable technology have been shown to be effective for promoting physical activity in cancer survivors generally, yet future interventions are needed that also address diet and alcohol consumption, and can be implemented into the UK NHS healthcare system (Qiu et al., 2023; Rodrigues et al., 2023). Future research is also required to develop lifestyle interventions for people from diverse ethnic backgrounds (Hailey et al., 2022), with much previous work focused on Caucasian women. In line with the ‘Making Every Contact Count’ behaviour change approach, it is also possible that a range of HCPs involved in breast care could potentially deliver aspects of a future intervention, such as radiographers for instance, at yearly post-cancer mammograms (Sinclair et al., 2019). In addition, other HCPs, such as physiotherapists, psychologists, social workers, cancer support workers or dieticians could be involved in the delivery of a future intervention, in terms of supporting behavioural change. Future research is required to explore these possibilities. Although the present study focused on breast cancer, it is likely that findings may be similar across other cancer types, which warrants further exploration.

### Strengths and limitations

Our study has usefully informed the design of future interventions, to address current unmet needs regarding lifestyle behaviours in UK breast cancer survivors. Due to the paucity of previous literature on alcohol consumption, we have also presented novel data exploring this topic, while also providing an in-depth insight into diet and exercise behaviours.

However, while we made efforts to advertise the study widely, the interview sample taken from the survey is all White females who were highly educated, so we are not able to generalise widely. Further research is needed to explore this topic in a more diverse sample. It should be noted that those selected to be invited for an interview were a purposive sample of people who had either a higher AUDIT score, or indicated that they had changed, either increased or reduced, their drinking since diagnosis, in order to understand more about alcohol consumption. We must be mindful therefore that this sample may have had more of a focus on, or need for, alcohol support than the general breast cancer population.

### Conclusions

Our findings highlight that personalised and empowering lifestyle-related support and information would be welcomed by people with breast cancer to promote exercise, diet and alcohol behavioural changes. Interventions are therefore needed, targeted at patients and also at HCPs to increase awareness regarding the benefits of a healthy lifestyle after breast cancer. These interventions should include alcohol consumption. Such interventions will enable people with breast cancer to make informed decisions regarding lifestyle changes and empower and support them during treatment and beyond.

## Supplemental Material

sj-docx-1-hpq-10.1177_13591053251336843 – Supplemental material for Lifestyle behaviour change following breast cancer: A qualitative exploration of experiences and unmet support and information needs Supplemental material, sj-docx-1-hpq-10.1177_13591053251336843 for Lifestyle behaviour change following breast cancer: A qualitative exploration of experiences and unmet support and information needs by Lauren Matheson, Lucy McGeagh, Julie Bennett, Emma L Davies, Jo Brett, Sara Matthews and Eila Watson in Journal of Health Psychology

sj-docx-2-hpq-10.1177_13591053251336843 – Supplemental material for Lifestyle behaviour change following breast cancer: A qualitative exploration of experiences and unmet support and information needs Supplemental material, sj-docx-2-hpq-10.1177_13591053251336843 for Lifestyle behaviour change following breast cancer: A qualitative exploration of experiences and unmet support and information needs by Lauren Matheson, Lucy McGeagh, Julie Bennett, Emma L Davies, Jo Brett, Sara Matthews and Eila Watson in Journal of Health Psychology

sj-docx-3-hpq-10.1177_13591053251336843 – Supplemental material for Lifestyle behaviour change following breast cancer: A qualitative exploration of experiences and unmet support and information needs Supplemental material, sj-docx-3-hpq-10.1177_13591053251336843 for Lifestyle behaviour change following breast cancer: A qualitative exploration of experiences and unmet support and information needs by Lauren Matheson, Lucy McGeagh, Julie Bennett, Emma L Davies, Jo Brett, Sara Matthews and Eila Watson in Journal of Health Psychology

sj-docx-4-hpq-10.1177_13591053251336843 – Supplemental material for Lifestyle behaviour change following breast cancer: A qualitative exploration of experiences and unmet support and information needs Supplemental material, sj-docx-4-hpq-10.1177_13591053251336843 for Lifestyle behaviour change following breast cancer: A qualitative exploration of experiences and unmet support and information needs by Lauren Matheson, Lucy McGeagh, Julie Bennett, Emma L Davies, Jo Brett, Sara Matthews and Eila Watson in Journal of Health Psychology

sj-docx-5-hpq-10.1177_13591053251336843 – Supplemental material for Lifestyle behaviour change following breast cancer: A qualitative exploration of experiences and unmet support and information needs Supplemental material, sj-docx-5-hpq-10.1177_13591053251336843 for Lifestyle behaviour change following breast cancer: A qualitative exploration of experiences and unmet support and information needs by Lauren Matheson, Lucy McGeagh, Julie Bennett, Emma L Davies, Jo Brett, Sara Matthews and Eila Watson in Journal of Health Psychology

sj-docx-6-hpq-10.1177_13591053251336843 – Supplemental material for Lifestyle behaviour change following breast cancer: A qualitative exploration of experiences and unmet support and information needs Supplemental material, sj-docx-6-hpq-10.1177_13591053251336843 for Lifestyle behaviour change following breast cancer: A qualitative exploration of experiences and unmet support and information needs by Lauren Matheson, Lucy McGeagh, Julie Bennett, Emma L Davies, Jo Brett, Sara Matthews and Eila Watson in Journal of Health Psychology

sj-docx-7-hpq-10.1177_13591053251336843 – Supplemental material for Lifestyle behaviour change following breast cancer: A qualitative exploration of experiences and unmet support and information needs Supplemental material, sj-docx-7-hpq-10.1177_13591053251336843 for Lifestyle behaviour change following breast cancer: A qualitative exploration of experiences and unmet support and information needs by Lauren Matheson, Lucy McGeagh, Julie Bennett, Emma L Davies, Jo Brett, Sara Matthews and Eila Watson in Journal of Health Psychology

sj-pdf-8-hpq-10.1177_13591053251336843 – Supplemental material for Lifestyle behaviour change following breast cancer: A qualitative exploration of experiences and unmet support and information needs Supplemental material, sj-pdf-8-hpq-10.1177_13591053251336843 for Lifestyle behaviour change following breast cancer: A qualitative exploration of experiences and unmet support and information needs by Lauren Matheson, Lucy McGeagh, Julie Bennett, Emma L Davies, Jo Brett, Sara Matthews and Eila Watson in Journal of Health Psychology

## Notes

1. Lifestyle behaviours and breast cancer: study protocol: Open Science Framework (osf.io/xabuj).
