# Supplementary material for: Lifestyle behaviour change following breast cancer: A qualitative exploration of experiences and unmet support and information needs
Source: J Health Psychol. 2025 Jun 11;31(3):1120–35. doi: 10.1177/13591053251336843 (PMC12949739; doi:10.1177/13591053251336843)
Supplement: sj-docx-1-hpq-10.1177_13591053251336843 – Supplemental material for Lifestyle behaviour change following breast cancer: A qualitative exploration of experiences and unmet support and information needs [file sj-docx-1-hpq-10.1177_13591053251336843.docx]

**Table 5: Additional exemplar quotes Theme 3**

| **Desire for empowering lifestyle advice – valuing “a strong call to arms”** |
| --- |
| The value of clear lifestyle advice  “At point of diagnosis say to people we’re gonna get you better, we’re gonna get rid of this cancer, and I’m sure you don’t want this to happen to you again, the reoccurrence rates are whatever they are in 10 years, but you can really help yourself from now on not to get this cancer to come back…and that is mainly down to you. So you need to get your weight under control, you need to start to exercise, you need to change your diet, you need to stop smoking, you need to start looking after yourself... Yeah, completely without judgment” (P7)  “I met a lot of people that were on chemo that would kind of say oh no, I’m going home to sit and eat some cake. I’m thinking why? You’re just sitting around making yourself feel sorry for yourself… but I think had they been given some advice to say just go for a little walk every day, I think it would be useful… I think anyone would benefit from these things ... They’re not the medical treatments, but the lifestyle treatments… Which is effectively what they are, are just as important …. actually all these lifestyle things… exercise, eating, alcohol” (P20) |
| The timing and delivery of desired advice  “Somehow getting information to people, and maybe it being at various points along the journey, because I think at some points you may be less able to take it on, whereas now I think I’d be in a much better place to make any changes that are needed, because I’m now through all the treatment” (P2)  “I do think a follow-up would be good, because that’s when, 6 to 12 months later, it is when you start to ask all of the questions, could I have done something to prevent it and what could I do differently and what changes could I make” (P19)  “I think for me, yes, at time of diagnosis, because I would want to make those changes” (P13)  “It’s difficult, I mean I think maybe a few weeks down from diagnosis, because you just wouldn’t take it in at the time” (P7) |
