# Supplementary material for: Lifestyle behaviour change following breast cancer: A qualitative exploration of experiences and unmet support and information needs
Source: J Health Psychol. 2025 Jun 11;31(3):1120–35. doi: 10.1177/13591053251336843 (PMC12949739; doi:10.1177/13591053251336843)
Supplement: sj-docx-2-hpq-10.1177_13591053251336843 – Supplemental material for Lifestyle behaviour change following breast cancer: A qualitative exploration of experiences and unmet support and information needs [file sj-docx-2-hpq-10.1177_13591053251336843.docx]

**Table 4: Additional exemplar quotes Theme 2**

| **Impact of lifestyle messaging from healthcare professionals** |
| --- |
| No advice received  “Nothing to say what I should and shouldn’t eat, and I don’t think I’ve ever had that, about what foods to avoid” (p11)  “I don’t remember getting that [lifestyle advice]. Yeah, I don’t remember that” (p3)  “I mean I don’t know whether it’s possible that some medical professionals, maybe they feel they can’t give advice about alcohol if they’re drinking themselves. That could be a factor, that people don’t feel they... if they themselves aren’t following that guidance. That’s a possibility.” (p2)  “I wasn’t given much, much on the health stuff. You kind of had to seek it out for yourself I suppose” (P8) |
| Weak advice  “I don’t think there was any specific from anybody. I think I probably asked, can I go running? And it was a yes, but it was in response to me asking them, it wasn’t them encouraging me to be active” (p13)  “And alcohol was mentioned as well...Just again my breast nurse. We never really had a conversation, she just said you know you need to watch what you’re drinking, your alcohol consumption” (p15) |
| Unhelpful self-care advice in lifestyle messaging  “So I remember asking my oncologist, and said, and I was looking at it for more vitamins and minerals and your diet, saying were there things I should or shouldn’t have, and she said oh no, if you want a glass of wine… That’s not what I was thinking of, she said you have one” P10  “When I went to my radiotherapy consultancy, I had a list of questions, because I was quite worried about it and scared, and one of them was can I drink alcohol. And the consultant laughed and said do you mean during radiotherapy? And I said well if I can I’ll have a glass of prosecco!... But he said no, it doesn’t matter, you can carry on” P8 |
| The salience of a passing comment  [**Interviewer: And then so you said you’ve changed since the consultant had a comment and everything, you’ve made some changes?]** "So now I would say I drink one bottle of wine a week, but I will have maybe two gin and tonics before dinner. I’m counting that as less alcohol, I don’t know whether it is or not. It’s certainly less calories anyway" (P17)  “So it seemed like everybody, all of the health professionals, were all saying take it easy, listen to your body, do what you can. Which I obviously took to be the least and didn’t do anything because I hadn’t done much before” (P4) |
