# Supplementary material for: Lifestyle behaviour change following breast cancer: A qualitative exploration of experiences and unmet support and information needs
Source: J Health Psychol. 2025 Jun 11;31(3):1120–35. doi: 10.1177/13591053251336843 (PMC12949739; doi:10.1177/13591053251336843)
Supplement: sj-docx-3-hpq-10.1177_13591053251336843 – Supplemental material for Lifestyle behaviour change following breast cancer: A qualitative exploration of experiences and unmet support and information needs [file sj-docx-3-hpq-10.1177_13591053251336843.docx]

**Table 3: Additional exemplar quotes Theme 1**

| **Impact of cancer and behaviour change complexities** |
| --- |
| Formation of new healthier habits  “Obviously drinking less. And since I’ve been on Tamoxifen I’ve noticed that drinking alcohol makes the hot flushes even worse. You kind of… It’s just not worth it sometimes. You like lose a whole night’s sleep. I might have had a drink but I know I’m not gonna sleep, so you have to go well hold on a minute. Yeah, I need to think do I want a drink or do I want to sleep. Usually I want to sleep! [Laughs]” (P5)  “I think I do more now. It’s only walking that I do. I find it’s really strange, if I do anything else, like sort of aerobic-type thing, I really feel quite spaced out and… I mean it’s weird.  So I do a lot of walking, and I’ll do at least 5K a day, I sometimes do 10, and it really helps my mental health” (P7)  “So yeah, there was a certain amount of processed ready meals I was eating, and I’m trying to really… I’ve really tried to increase my fruit and vegetable intake... But I mean I’m very motivated, and I, when I found out I had cancer I bought all these books, recipe books and books about breast cancer, so I’m quite sort of geared up about what is good to eat and not” (P1)  Reasons for lack of behavioural changes  “I would say it’s probably gone back to, over time it’s built up. But again I can, I suppose I can take it or leave it [alcohol]. I do try. Again it’s usually back to the Friday and Sunday evening, but I would try to cut back, I’ll have smaller glasses. But then you know, over Christmas or holidays or something we will drink more. So yes, so probably drinking the same” (p13)  “So I would say my diet hasn’t really changed. I think I’m fairly good on the diet front.” (P15) |
