# Supplementary material for: Lifestyle behaviour change following breast cancer: A qualitative exploration of experiences and unmet support and information needs
Source: J Health Psychol. 2025 Jun 11;31(3):1120–35. doi: 10.1177/13591053251336843 (PMC12949739; doi:10.1177/13591053251336843)
Supplement: sj-docx-4-hpq-10.1177_13591053251336843 – Supplemental material for Lifestyle behaviour change following breast cancer: A qualitative exploration of experiences and unmet support and information needs [file sj-docx-4-hpq-10.1177_13591053251336843.docx]

**Table 2: Summary of themes**

| Theme | Sub-theme |
| --- | --- |
| Impact of cancer and behaviour change complexities | Formation of new healthier habits |
|  | Reasons for lack of behavioural changes |
| Impact of lifestyle messaging from healthcare professionals | No advice received |
|  | Weak advice |
|  | Unhelpful self-care advice in lifestyle messaging |
|  | The salience and impact of a passing comment |
| Desire for empowering lifestyle advice – valuing “a strong call to arms” | The value of clear lifestyle advice |
|  | The timing and delivery of desired advice |
| Shaping future lifestyle interventions | Multiple modes of delivery and a ‘one stop shop’ |
|  | Personalised support from a trustworthy source |
|  | Social support and emphasis on the benefits of lifestyle changes |
