# Supplementary material for: Lifestyle behaviour change following breast cancer: A qualitative exploration of experiences and unmet support and information needs
Source: J Health Psychol. 2025 Jun 11;31(3):1120–35. doi: 10.1177/13591053251336843 (PMC12949739; doi:10.1177/13591053251336843)
Supplement: sj-docx-5-hpq-10.1177_13591053251336843 – Supplemental material for Lifestyle behaviour change following breast cancer: A qualitative exploration of experiences and unmet support and information needs [file sj-docx-5-hpq-10.1177_13591053251336843.docx]

**Lifestyle behaviours after cancer – Coding Framework**

| **Code** | **Sub-code** | **Description** |
| --- | --- | --- |
| **Behavioural context**  **and**  **changes**  **in behaviour** | Pre cancer alcohol consumption | This could include identity, general description of their prior behaviour and general context – not necessarily cancer related, co-morbidities, young family, aging etc |
|  | Pre cancer diet and nutrition | This could include identity, general description of their prior behaviour and general context – not necessarily cancer related, co-morbidities, young family, aging etc |
|  | Pre cancer exercise levels | This could include identity for example ‘I’ve always been fit and exercised lots’, general description of their prior behaviour and general context – not necessarily cancer related, co-morbidities, young family, aging etc |
|  | Changes to alcohol consumption | This could include increase, decrease, change in type of alcohol or where they drink and who with, including reasons why – including un-cancer related issues |
|  | Changes to exercise | This could include increase, decrease, change in type of exercise or where they exercise, intensity, who with, including reasons why – including un-cancer related issues |
|  | Changes to diet and nutrition | This could include increase, decrease, change in type of food or where they are eating, what they are eating, who they are eating with, including reasons why – including un-cancer related issues |
|  | No change to alcohol consumption since diagnosis | Any suggestion that they have not made changes to alcohol consumption since diagnosis, and reasons why |
|  | No change to exercise since diagnosis | Any suggestion that they have not made changes to exercise levels or type since diagnosis, and reasons why |
|  | No change to diet and nutrition since diagnosis | Any suggestion that they have not made changes to diet and nutrition, or food in any way, since diagnosis and reasons why |
|  | Changes you wish to make to alcohol consumption | This may relate to drinking more or less, or changes to the type of drink, how they drink, where, or who they drink with |
|  | Changes you wish to make to exercise | This may relate to increasing or decreasing exercise levels, or changing the type of exercise, or who they exercise with |
|  | Changes you wish to make to diet and nutrition | This may relate to any food, diet or nutrition related changes |
|  | Impact of cancer diagnosis or treatment on lifestyle | e.g., it was a huge shock, expected the diagnosis, difficult to talk to family about it, worry about recurrence – only things relating to lifestyle behaviours |
|  | Impact of cancer diagnosis or treatment on anything else - non-lifestyle related | e.g., it was a huge shock, expected the diagnosis, difficult to talk to family about it, worry about recurrence- only relating to things that are non-lifestyle behaviours |
| **Guidance, knowledge**  **and**  **belief**  **about behaviour change** | Official recommendations for alcohol consumption, including government guidance | e.g. drinking a maximum of 14 units of alcohol – this could include general knowledge or awareness of official gov guidelines, for the general population or cancer specific – the information doesn’t need to be correct |
|  | Official recommendations for exercise levels, including government guidance | e.g. 30 minutes 5 times a week this could include general knowledge or awareness of official gov guidelines, for the general population or cancer specific – the information doesn’t need to be correct |
|  | Official recommendations for diet and nutrition, including government guidance | e.g. eating 5 fruit and veg a day – this could include general knowledge or awareness of official gov guidelines, for the general population or cancer specific – the information doesn’t need to be correct |
|  | Beliefs about alcohol and cancer | This could include alcohol related causes of cancer or causes of recurrence, misconceptions and knowledge. This could include sources of knowledge or beliefs, such as lay advice from peers, family, websites etc – not including HCP or gov guidance (code above) |
|  | Beliefs about exercise and cancer | This could include exercise related causes of cancer or causes of recurrence, misconceptions and knowledge. This could include sources of knowledge or beliefs, such as lay advice from peers, family, websites etc – not including HCP or gov guidance (code above) |
|  | Beliefs about diet and nutrition and cancer | This could include diet or nutrition related causes of cancer or causes of recurrence, misconceptions and knowledge. This could include sources of knowledge or beliefs, such as lay advice from peers, family, websites etc – not including HCP or gov guidance (code above) |
|  | Beliefs about causes of cancer - either general lifestyle or not lifestyle related | This could include beliefs around causes of cancer or recurrence that are around **general** lifestyle behaviours (e.g. not specific to diet, exercise or alcohol), or causes of cancer that are to do with anything else that is non-lifestyle related e.g. bad luck, genetics etc. |
|  | Advice or support from professionals about alcohol | This could include any information, conversations, leaflets or referrals relating to alcohol and cancer – correct or not and can also include a lack of information given. Also includes appropriateness of timing of this information. Include where HCP has advised about gov or other official guidance |
|  | Advice or support from professionals about exercise | This could include any information, conversations, leaflets or referrals relating to exercise and cancer – correct or not and can also include a lack of information given. Also includes appropriateness of timing of this information. Include where HCP has advised about gov or other official guidance |
|  | Advice or support from professionals about diet and nutrition | This could include any information, conversations, leaflets or referrals relating to food, diet, nutrition and cancer – correct or not, and can also include a lack of information given. This could include support from organisations such as slimming world. Also includes appropriateness of timing of when information given. Include where HCP has advised about gov or other official guidance |
|  | Advice or support from professionals regarding general lifestyle or anything else that is not diet, exercise or alcohol related | This could include anything about general advice or support that is non-specific e.g. not relating to alcohol, diet, nutrition or exercise. For example, advice (or lack of) regarding generally being healthier, and also advice (or lack of) to anything else that may be relevant to health such as weight or mental health for example. |
| **Barriers**  **and**  **facilitators**  **to**  **actual**  **or**  **desired behaviour**  **and**  **behaviour change** | Experienced and perceived benefits of alcohol | e.g. coping mechanisms, short term benefits (feeling better, sleeping better, lose weight, lower cholesterol etc), long term benefits (health implications reduce risk of recurrence) of alcohol |
|  | Experienced and perceived benefits of exercise | e.g. coping mechanisms, short term benefits (feeling better, sleeping better, lose weight, lower cholesterol etc), long term benefits (health implications reduce risk of recurrence) of exercise |
|  | Experienced and perceived benefits of diet and nutrition | e.g. coping mechanisms, short term benefits (feeling better, sleeping better, lose weight, lower cholesterol etc), long term benefits (health implications reduce risk of recurrence) of specific diet and nutrition |
|  | Experienced and perceived benefits of changing alcohol levels | e.g. short-term benefits (feeling better, sleeping better, lose weight, lower cholesterol etc), long term benefits (health implications reduce risk of recurrence), e.g. ‘whatever it takes for the cancer not to return’, of changing alcohol levels |
|  | Experienced and perceived benefits of changes to exercise | e.g. short-term benefits (feeling better, sleeping better, lose weight, lower cholesterol etc), long term benefits (health implications reduce risk of recurrence), e.g. ‘whatever it takes for the cancer not to return’, of changes to exercise |
|  | Experienced and perceived benefits of changes to diet and nutrition | e.g. short-term benefits (feeling better, sleeping better, lose weight, lower cholesterol etc), long term benefits (health implications reduce risk of recurrence), e.g. ‘whatever it takes for the cancer not to return’, of changes to diet and nutrition |
|  | Experienced and perceived negatives of alcohol or barriers to drinking alcohol | e.g., physical symptoms either from cancer treatment or hangovers for example, related to alcohol |
|  | Experienced and perceived negatives of exercise or barriers to doing exercise | e.g., physical symptoms either from cancer treatment or aging causing aches and pains for example, related to exercise |
|  | Experienced and perceived negatives of specific food, diet or nutrition | e.g., physical symptoms either from cancer treatment or how certain foods make them feel or how much they cost or time to cook from scratch for example, related to specific diet and nutrition |
|  | Experienced and perceived negatives of changing alcohol levels or barriers to changing drinking | e.g., physical symptoms either from cancer treatment or not having hangovers for example, related to changes to alcohol |
|  | Experienced and perceived negatives of changing exercise levels or barriers to changing exercise levels | e.g., physical symptoms either from cancer treatment related to the change, related to changes to exercise |
|  | Experienced and perceived negatives of changing what is being eaten - specific food, diet or nutrition | e.g., physical symptoms either from cancer treatment related to the change, related to changes to specific diet or nutrition |
|  | Facilitators to changing alcohol consumption – | e.g., focus on what is achievable, goal setting, opportunistic situations, adapting to circumstances, this could also include contextual factors related to changing alcohol consumption |
|  | Facilitators to changing exercise levels | e.g., focus on what is achievable, goal setting, opportunistic exercise, adapting to circumstances (can’t do what I use to, but can do something), use of apps/wrist monitors, social support (e.g. doing exercise with others) this could also include contextual factors such as being retired so having more time, related to changing exercise levels |
|  | Facilitators to changing diet and nutrition | e.g., focus on what is achievable, goal setting, opportunistic healthy eating, adapting to circumstances (can’t do what I use to, but can do something), this could also include contextual factors such as being retired so having more time, related to diet and nutrition |
| **Intervention ideas**  **(Any**  **discussion about experiences**  **of,**  **or**  **suggestions**  **for,**  **an intervention, including**  **their reasoning)** | Mode of intervention | Any discussion about face to face, groups, online, apps and digital support including wearable technology, preference for digital or paper |
|  | Motivation to change | Include what they think may help them or others, for example, cancer as a scare, shock tactics, people need to want to change, people need to take responsibility for themselves, equal emphasis on short term benefits and long-term benefits, sense of achievement/praise along the way, whatever it takes for cancer not to return, fear of recurrence |
|  | Consideration of financial implications | Anything relating to cost of living or finance |
|  | Empowering people | Anywhere they talk about what empowers them, or how to empower others |
|  | Ideal time for support or information | E.g. at diagnosis, post treatment. This can include their actual experience of having or not having support/information at the right time |
|  | Techniques to help with behaviour change | Include things such as making small changes or setting achievable goals e.g. 30 mins a day, needing to fit with current lifestyle, opportunistic E.g. exercise (walk to shops, leave walking boots in car) |
|  | Sources of information | Any discussion about where info should come from e.g. HCP, scientific refs, stats, quotes from other women |
|  | Social support component | e.g., group support, zoom catchups, forums or preference not to have interaction with others |
|  | Tailored support | Anything relating to personalised or tailored support |
|  | Existing ‘Moving forward and other courses’ | This could include their views of or experience of existing interventions or programmes, or apps for example |
|  | Key content | Any suggestions of what an intervention should include, e.g. providing information |
