# Supplementary material for: Lifestyle behaviour change following breast cancer: A qualitative exploration of experiences and unmet support and information needs
Source: J Health Psychol. 2025 Jun 11;31(3):1120–35. doi: 10.1177/13591053251336843 (PMC12949739; doi:10.1177/13591053251336843)
Supplement: sj-docx-6-hpq-10.1177_13591053251336843 – Supplemental material for Lifestyle behaviour change following breast cancer: A qualitative exploration of experiences and unmet support and information needs [file sj-docx-6-hpq-10.1177_13591053251336843.docx]

**Interview Topic Guide**

**Breast cancer diagnosis background and current (if any) hospital visits, treatment and side effects**

**Current Lifestyle:**

1. **Exercise -** before, during and after treatment (include changes, types, location and frequency of exercise)

- Explore any changes **–** how & what influenced them, how easy/difficult to make?
- If no changes – were they considered, what hindered change?
- Perceived benefits of exercising - feelings before, during and after exercising
- Perceived negatives, challenges, barriers
- Desire to change? What would help?
- Any advice, support or information about exercise received? Where from, when and how?
- What was the advice, views on advice?
- If none received, was it wanted? When, how, what aspect of lifestyle in particular?
- Is it wanted now?

1. **Alcohol consumption -** before, during and after treatment (frequency, quantity, type, where and when (social?)

- What is a typical drinking occasion now, benefits/negatives of alcohol?
- Changes since diagnosis – how, why?
- If less now – a conscious/deliberate change? Easy/difficult to change, why?
- If more now - a conscious/deliberate change? Why?
- If no change – any thoughts of change? What hindered change?
- Any advice, support or information about alcohol received - where from, when and how?
- What was the advice, views on advice?
- If none received, was it wanted? When, how, what aspect of lifestyle in particular?
- Desire to change? How? What would help?

1. **Nutrition –** current eating habits – comparison with before diagnosis. Eating habits before, during and after breast cancer treatment

- Changes made – how, why?
- If change to more healthy diet – was it difficult? why?
- If no change - any thoughts of change? What hindered change?
- What types of foods in diet, what avoided?
- Perceived benefits of eating healthy? Any negatives?
- Desire to change? What would help?
- Any advice, support or information about nutrition received - where from, when and how?
- What was the advice, views on advice?
- If none received, was it wanted? When, how, what?
- Is it wanted now?

1. **Smoking** (if ‘smoker’ indicated on survey) - habits before, during and after treatment – frequency, quantity, type, when and where (social?), why, benefits/negatives?

- Any changes – how, why?
- If less – a conscious/deliberate change? Easy/difficult to change, why?
- If no change **-** any thoughts of change? If tried - difficult? Why? If not, what hindered changes?
- Desire to change? How?
- Any advice, support or information about nutrition received - where from, when and how?
- What was the advice, views on advice?
- If none received, was it wanted? When, how, what?
- Is it wanted now?

**For all of the behaviours:**

- Any knowledge of specific guidelines for lifestyle behaviours after breast cancer? wanted? when?
- Help to make changes? (*refer to* *responses given),* use examples: information, education, time or money, social support, motivation, emotional support.

**Interventions** (study aims to identify how best to support people who would like to change their lifestyle behaviours):

- What sort of things would be helpful/unhelpful? Provide suggestions of interventions and ask for feedback.
- Ask for other ideas, include need for social aspect, views on timing.
