# Supplementary material for: Lifestyle behaviour change following breast cancer: A qualitative exploration of experiences and unmet support and information needs
Source: J Health Psychol. 2025 Jun 11;31(3):1120–35. doi: 10.1177/13591053251336843 (PMC12949739; doi:10.1177/13591053251336843)
Supplement: sj-docx-7-hpq-10.1177_13591053251336843 – Supplemental material for Lifestyle behaviour change following breast cancer: A qualitative exploration of experiences and unmet support and information needs [file sj-docx-7-hpq-10.1177_13591053251336843.docx]

**Table 6: Additional exemplar quotes** **for Theme 4**

| **Shaping future lifestyle interventions** |
| --- |
| Multiple modes of delivery for a “one stop shop” intervention  “I suppose an app. I do use the apps, yeah. The app is good, but I also like a book, I like to be able to make notes” (P13)  “I suppose maybe in a written form might be useful. So you can just look at it in your own time, take from it what you want. I mean I wouldn’t mind if they said it to me, if it was spoken to me it wouldn’t bother me, but it might be better to just take away... So digital would be fine, if I were sent an e-mail with it that would work absolutely for me” (P7) |
| Personalised support from a trustworthy source  “I think certainly alcohol I think a medical person is much better placed to be able to advise you on the effects that’s going to have on you….so I think a really good resource that you can trust, I mean that would be lovely if something like that existed, and then all the health professional had to do was to just even direct you to that and say look, if you’re interested in any sort of ideas of changing behaviours, if you feel you want to or need to then look, this is a really good resource, this can answer your questions” (p16)  “If you could see that you had this plan, and that there was something at the end, I don’t want to be two stone heavier, I don’t want to be tired, I don’t want to have to buy new clothes because I can’t fit in them… goal oriented, that would have really helped me” (P4)  “I think that breast cancer nurse would be in the best place to give you that information” (P10)  “I think certain scenarios it would need to be with a healthcare professional… Because there’s so many, you could look on the internet and just find all sorts of terrible advice, so I think it has to come from a healthcare professional” (P7) |
| Social support and emphasis on the benefits of lifestyle changes  “Maybe a list of support groups that they could go to if they wanted to, nationally and locally.” (P1)  “Although no-one else has got my stage of cancer, they’ve [others in exercise group] all got exercise in common, so instead of all you’ve got in common is cancer, you’ve actually got other things, and it feels a very positive thing to have in common with other people, so I think that’s become really important to me” (P16)  “I think exercise is potentially the one that could make the best difference to people… We need to be presenting them with the information as to why it’s good for them” (P16)  “So you’ve got to make people realise why lifestyle changes are so important, and then kind of provide things to support them to make those changes” (P19) |
